# Effects of Cadmium Stress on Root and Root Border Cells of Some Vegetable Species with Different Types of Root Meristem

**DOI:** 10.3390/life12091401

**Published:** 2022-09-09

**Authors:** Yingming Feng, Huanxiu Li, Xianshi Zhang, Xuewen Li, Jie Zhang, Lei Shi, Xingyun Chen, Wei Nong, Changquan Wang, Sergey Shabala, Min Yu

**Affiliations:** 1 International Research Centre for Environmental Membrane Biology, Department of Horticulture, Foshan University, Foshan 528000, China; 2 College of Resources and Environment, Huazhong Agricultural University, Wuhan 430070, China; 3 College of Horticulture, Sichuan Agricultural University, Chengdu 611130, China; 4 College of Resources and Environment, Sichuan Agricultural University, Chengdu 611130, China; 5 Tasmanian Institute of Agriculture, University of Tasmania, Hobart, TAS 7001, Australia; 6 School of Biological Science, University of Western Australia, Crawley, WA 6009, Australia

**Keywords:** root apical meristem, cadmium toxicity, root border cells, vegetables

## Abstract

Cadmium is one of the most toxic heavy metals and can be easily absorbed by plants, affecting root growth. Root border cells (RBCs), that are located in the periphery of the root cap and originate from the root cap meristem, represent a convenient tool to study the toxic effects of Cd on root performance. In this work, vegetables with contrasting types of root apical meristem (RAM) organizations were used. The open RAM organizations included pea and cucumber, and the closed RAM organizations included tomato, chili, and eggplant. The number of RBCs were significantly higher in the species possessing open RAM organization: pea (11,330 cells per root) > cucumber (8200) > tomato (2480) > eggplant (1830) > chili (1320). The same trend was observed for cell viability: pea (61%) > cucumber (59%) > tomato (49%) > eggplant (44%) > chili (42%). Pea and cucumber had higher relative radicle elongation rates and a lower increase in stress-induced accumulation of malondialdehyde (MDA), making them more resistant to Cd stress than the vegetables with close RAM organization. Under Cd treatment, the number and viability of RBCs in vegetables with both types of RAM organization were significantly decreased. However, the decreasing ratio of the number and viability of RBCs in pea and cucumber was higher than in tomato, chili, and eggplant. Taken together, the plants with the open-type RAM are more tolerant to Cd, and it can be speculated that the cadmium tolerance of the vegetables may be correlated with the number and viability of RBCs in response to cadmium stress.

## 1. Introduction

Cadmium is one of the most toxic heavy metal elements, which is highly mobile and can be easily absorbed by plants [1,2]. Cadmium could seriously affect the production and quality of the crops, and further affect human health while entering the food chain [3,4]. Cadmium is highly toxic to the root system of crops, especially to the root apex [5].

The root tip consists of a distal root cap and a proximal root apical meristem (RAM) from which all primary root tissues are derived [6]. A population of self-perpetuating cells called initials is located at the pole of the RAM that supplies cells for the meristem. Depending on the shape of the initials and cell division activity, two types of RAM are distinguished. If all the root tissues and root cap share the same initials, the root belongs to the open-type RAM. However, if the pericycle, cortex, root cap, and epidermal cells have their own initials, such roots belong to the closed-type RAM [6]. Despite the significant difference in root anatomical organization, very few reports are available dealing with the physiological and ecological significance of root tip meristem types.

Root border cells (RBCs) are a group of cells originating from the root cap meristem, which are developed by genetic regulation and play a variety of biological functions [7,8,9]. Being anatomically different from other root tip cells, RBCs may possess some unique functional features, such as the production and release of specific substances, regulating the rhizosphere environment, reducing root mechanical resistance, and protecting the root tip [10,11,12]. In addition, they also play important roles in avoiding or mitigating adverse environmental and biotic stresses such as pests and diseases, heavy metals, and moisture and temperature extremes. It has been found that RBCs possess protective effects against the apical aluminum toxicity, but the physiological and ecological significance of RBCs in the rhizosphere is still unclear. Specifically, the roles of RBCs in responding to rhizosphere stress factors such as cadmium toxicity have not been reported. Since the number and viability of RBCs in the plants with open- and closed-RAM are quite different, we hypothesize that the responses of plants with different types of RAM to Cd stress might be related to the production of RBCs and their responses to Cd.

In the present study, the aeroponics system was used to study the response of the root and RBCs of vegetables with an open and closed root-tip meristem to cadmium, in order to establish a possible causal relationship between RAM type and plant tolerance to heavy metals. It is concluded that the above difference is due to the difference in the absolute number of RBCs (larger in open-RAM species) that may operate as buffers protecting sensitive cells in the root apex against Cd toxicity.

## 2. Materials and Methods

### 2.1. Plant Materials

According to the different types of RAM organization, the following dicotyledonous vegetable crop species were used: plants with open-type RAM organization—pea (*Pisum sativum* L.) (cv Zhongwan 5 and Zhongwan 6) and cucumber (*Cucumis sativus* L.) (cv Jinmantian and Huayou 1); plants with closed-type RAM organization—chili (*Capsicum frutescens* L.) (cv Guangjao 1 and Zhongjiao 5); eggplant (*Solanum melongena* L.) (cv Yuanfeng zihongqie); tomato (*Solanum lycopersicum* L.) (cv Dongfanghong). The above materials were provided by Department of Horticulture, Foshan University (International Research Centre for Environmental Membrane Biology, Foshan, China).

### 2.2. Sterilization and Germination of the Seeds

Seeds were sterilized using 95% ethanol (Guangzhou Chemical Reagent Factory, Guangzhou, China) for 10 min, followed by 7.5% NaClO (Guangzhou Chemical Reagent Factory, Guangzhou, China) for 25 min, and then washed with sterile Mili-Q water six times, 5 min per washing, to remove the floating seeds and the seeds infected with pests. After then, the seeds were soaked with 0.5 mmol/L CaCl_2_ (Chengdu Cologne Chemical Reagent Factory, Chengdu, China) at 25 °C in a dark room. Pea seeds were soaked for 8 h, and cucumber, chili, eggplant, and tomato seeds were soaked for 24 h. The soaked seeds were applied to aeroponics culture for 48–72 h using 0.5 mmol/L CaCl_2_ (spraying 45 s per 5 min) in the aeroponics culture box specific for RBCs cultivation [13].

### 2.3. Measure the Number and Viability of RBCs

When the roots grew to 3 cm long, RBCs were collected and tested for their quantity and viability. Roots were gently cut and immersed in a tube filled with distilled water. RBCs were shed into the water by vigorous shaking. Trypan blue staining was used to test cell number and viability. 100 μL cell collection fluid was added to 100 μL 0.5% trypan blue stain solution (Sigma-Aldrich, St. Louis, MO, USA) and kept at room temperature for 15 min. Then 5 μL of the above mixture was taken and spread on a glass slide. A total of 10 tubes were collected and each tube was measured 3 times. The number of living cells (unstained) and dead cells (stained) was counted under the microscope.

### 2.4. Treatments

Uniformly grown seedlings were selected and transferred to another aeroponics culture incubator (0.5 mM CaCl_2_, pH 5.5). The different concentrations of CdCl_2_ (Guangzhou Chemical Reagent Factory, Guangzhou, China) solutions (0, 0.25, 0.5, 1, 2 mmol/L) were added to the culture solution, respectively. After further culturing for 48 h, the root length was measured. Root tips were excised and washed with double distilled water (DDW). After drying surface water, the content of malondialdehyde and cadmium was measured. Each treatment was repeated four times.

### 2.5. Measurement of Root Length, Malondialdehyde (MDA), and Cd Content

The root length was measured by a ruler with an accuracy of 0.01 cm, and 20 pieces of roots were measured for each treatment. The relative root length (% control) was calculated as an increment in a root length under treatment conditions divided by a similar value under the control condition (×100%).

The content of MDA was determined using the thiobarbituric acid method. The 2 cm long apical root segments were cut and washed with DDW three times. Roots were blotted and then 0.2 g fresh sample was ground. The thiobarbituric acid method was used to determine the MDA content [14]. The relative MDA content was calculated (as % control). The samples were then dried under 60 °C for 2–3 days and digested using HNO_3_. The cadmium content was measured using atomic absorption spectrophotometer.

### 2.6. Data Processing

The statistical analysis of the results was carried out using Excel2013 (Microsoft, Washington, DC, USA) and SAS8.1 (SAS Institute, Cary, NC, USA) software. The data were shown as average value ± SD. The significance of the difference was analyzed using Duncan’s test under *p* < 0.05 level.

## 3. Results

### 3.1. Differences in the Quantity and Viability of RBCs among Different Vegetable Species

**Figure 1:**
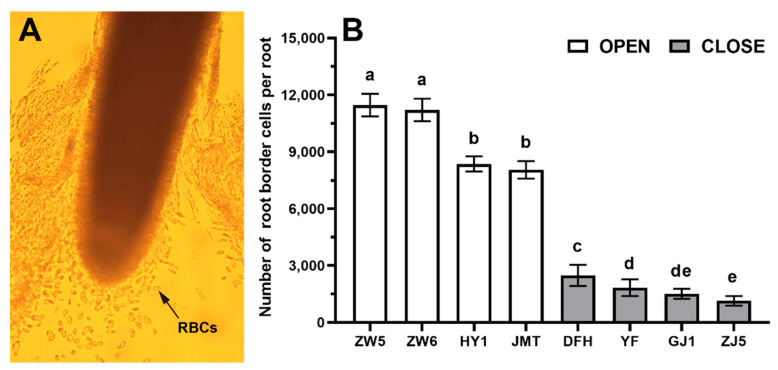
The number of RBCs in different vegetable species. (**A**) Location of RBCs in the root tip (pea as an example); (**B**) Number of RBCs per root. Data points and error bars represent mean and SD of 30 collective biological replicates. OPEN = open RAM organization; CLOSE = closed RAM organization. ZW5 = Zhongwan 5; ZW6 = Zhongwan 6; JMT = Jinmantian; HY = Huayou 1; GJ1 = Guangjao 1; ZJ5 = Zhongjiao 5; YF = Yuanfeng Zihongqie; DFH = Dongfanghong. Data labeled with different letters is significantly different at *p* < 0.05 level (Duncan’s test).

The number of RBCs was significantly different among different vegetable species (Figure 1). Among them, the number of RBCs in peas was the biggest, reaching values around 11,330 cells per root (CPR). Chili plants had the least number of RBCs, only around 1320 CPR. The number of RBCs in different species was as the following order: pea > cucumber > tomato > eggplant > chili. No significant difference was found between different varieties within the same species. The number of RBCs was much larger in the vegetables with an open-RAM organization than in the species with a closed-RAM organization. Among the vegetables with open-RAM organization, the Zhongwan 5 contained the largest number of RBCs, while the Jinmantian contained the smallest number, about 8050 CPR. In the case of species with the closed-RAM organization, the number of RBCs in Dongfanghong was the highest, 2481 ± 557 CPR, while in Zhongjiao 5 was the smallest, only 1137 ± 253 CPR.

**Figure 2:**
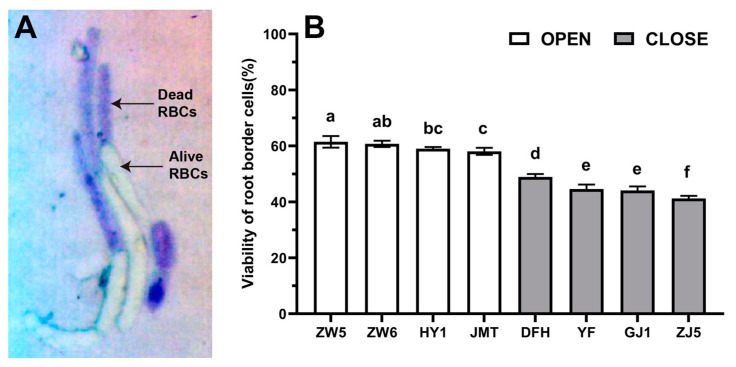
The viability of RBCs in different species. (**A**) Differentiation of alive and dead RBCs after trypan blue staining method (pea as an example); (**B**) Viability of RBCs. Data points and error bars represent mean and SD of 30 collective biological replicates. Data labeled with different letters is significantly different at *p* < 0.05 level (Duncan’s test).

The viability of RBCs was measured by the trypan blue staining method (Figure 2A). The survival rate of the RBCs varied among different species of vegetables (Figure 2B). The average survival rate of RBCs was about 61% in peas, the highest among all species, while it was only about 42% in the chili. There was no significant difference between different varieties of the same species except Guangjao 1 and Zhongjiao 5 of chili. The survival rate of RBCs in different species ranked as follows: pea > cucumber > tomato > eggplant > chili, consistent with the quantity of RBCs. The survival rate of RBCs was significantly higher in the species with the open-RAM organization than in their closed-RAM counterparts. Among the species with an open-RAM organization, the RBCs from Zhongwan 5 showed the highest viability (61.46 ± 2.09%), while that from Jinmantian possessed the lowest viability (58.08 ± 1.28%). In the case of species with closed-RAM organization, the survival rate of RBCs in Dongfanghong was the highest (48.93 ± 1.06%) and the lowest was in Zhongjiao 5 (41.23 ± 0.93%).

### 3.2. Effects of Cadmium Treatment on the Growth of Root-Tip of Different Vegetables

There was a great difference in the radicle elongation among different species. Cadmium exposure significantly inhibited the radicle growth, in a dose-dependent manner. The relative root elongation of peas and cucumbers was higher than tomatoes and chili (Table 1). Under the same concentration of Cd treatment, the relative root length (RRL) of vegetables with open RAM was larger than that with closed RAM, indicating that the roots of vegetables with closed RAM may be more vulnerable to cadmium toxicity. The radicles of vegetables with an open RAM were more tolerant to Cd than that of species with the close RAM.

### 3.3. The Effects of Cd Treatments on MDA Contents in Roots of Vegetables

With the increasing concentration of Cd treatment, the MDA content was significantly increased, in a dose-dependent manner, in the root tips of all species (Table 2).

Also proportional was an increase in Cd concentration in roots. Since the base content of MDA in the roots of different species was different, the relative increasing rate was used to compare the variation of root MDA content between different vegetable species. Under the same concentration of Cd treatment, the relative increase of the MDA content in the species with an open RAM was less than that in the species with the closed RAM, which demonstrated that the membrane lipid peroxidation in species with closed RAM was more pronounced, thus producing more MDA.

### 3.4. The Effects of Cd Concentrations on the Number of RBCs of Pea

**Figure 3:**
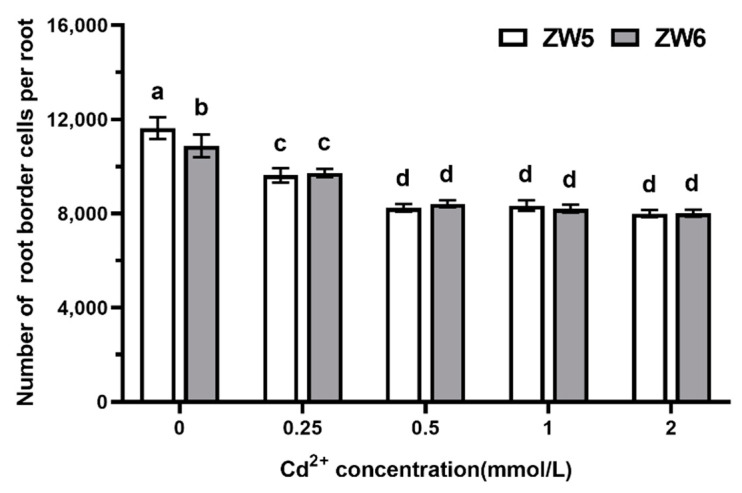
The effects of Cd concentrations on the number of RBCs of pea. Data are mean ± SD (n = 30). Data labeled with different letters is significantly different at *p* < 0.05 level (Duncan’s test).

The effects of Cd on the number and viability of RBCs were studied using peas as a model species. As can be seen from Figure 3, the number of RBCs was reduced as Cd concentration increased. The observed effect was dose-dependent in the range of 0–0.5 mmol/L, and the number of RBCs showed no further reduction when the CdCl_2_ concentration reached above 0.5 mmol/L. No significant difference between the two varieties tested was observed.

**Figure 4:**
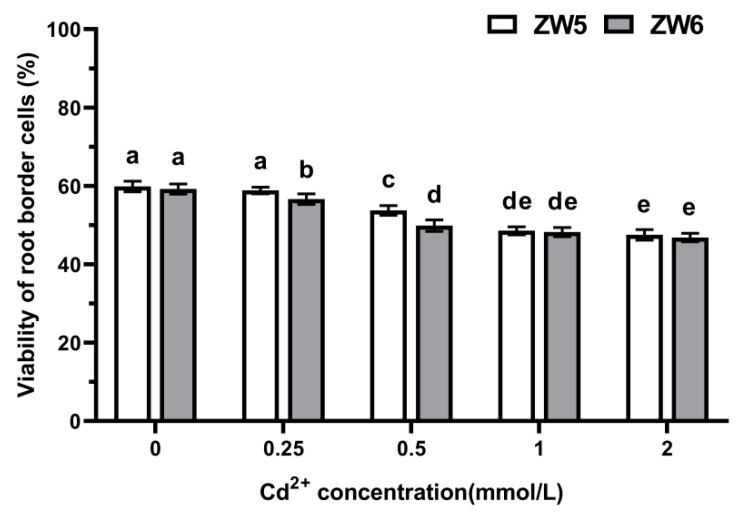
The effects of Cd concentrations on the viability of RBCs of pea. Data are mean ± SD (n = 30). Data labeled with different letters is significantly different at *p* < 0.05 level (Duncan’s test).

The survival rate of RBCs decreased with the increase in Cd concentration in a dose-dependent manner (Figure 4), with no obvious difference between the two cultivars.

### 3.5. The Response of RBCs to Cadmium

**Figure 5:**
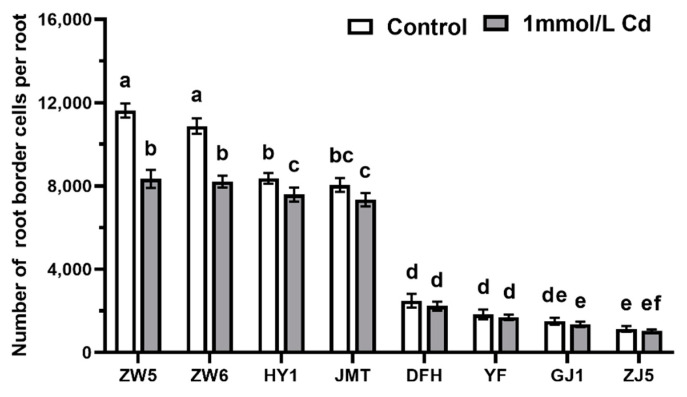
The effects of Cd on the number of RBCs of vegetables. Data are mean ± SD (n = 30). Data labeled with different letters is significantly different at *p* < 0.05 level (Duncan’s test).

The numbers of RBCs in different species decreased under 1 mmol/L CdCl_2_ treatment compared with the control conditions but the significant (at *p* < 0.05) difference was observed only in species with an open RAM such as peas (Zhongwan 5 and Zhongwan 6) and cucumbers (Huayou 1 and Jinmantian). In species with closed RAM (chili, eggplant, and tomatoes) the reduction was not statistically significant (Figure 5).

**Figure 6:**
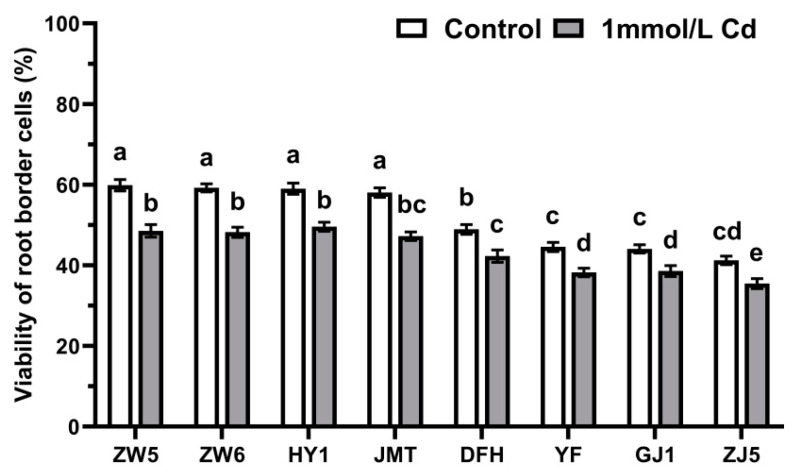
The effects of Cd on the viability of RBCs of vegetables. Data are mean ± SD (n = 30). Data labeled with different letters is significantly different at *p* < 0.05 level (Duncan’s test).

The viability of RBCs in various species significantly decreased after being treated with 1 mmol/L CdCl_2_ (Figure 6). Again, this decrease was much stronger in species with open RAM compared with those with closed RAM.

## 4. Discussion

By analyzing 35 different species, Groot et al. [6] reported 23 different anatomical structures in their roots. According to the original cell morphology, cell division activity, and tissue partitioning features, meristems can be roughly divided into three types: basic-open RAM, intermediate-open RAM, and closed RAM. It has been speculated that there is a strong relationship between the generation, quantity and survival rate of RBCs and the meristem structure types. Feng et al. [7] analyzed the number and activity of RBCs of 30 varieties of 11 different species including *Solanaceae*, *Leguminosae*, *Cucurbitaceae*, *Geamineae*, *Laguminosae*, and *Pinaceae*. By combining Hawes and Groot’s study [6,7], Hamamoto et al. [15] found that the number and activity of RBCs in open (including basic open-and semi-open) type species were higher than that in closed type species. Thus, the number and viability of RBCs may be highly related to the structure of meristematic tissue.

In the present study, we studied the number and activity of RBCs from several vegetable species. Our study confirmed that the number and survival rate of RBCs from open-type peas and cucumbers was significantly higher than in closed-type tomatoes, chili, and eggplants. However, the number of RBCs detected by our method was significantly higher than that from other kinds of literature, which may be due to the difference in growth conditions and, specifically, the capability of the aeroponics culture to keep a complete set of RBCs. Thus, we believe that using aeroponics culture to study the number of RBCs is more accurate and reliable than other methods. In addition, the discrepancy in the survival rate of RBCs between open-and closed-type apical meristem is smaller than in other reports, which may be because the survival rate was reflected by cell membrane permeability measured by trypan blue staining.

The inhibition of root elongation is the most visible symptom of Cd toxicity in plants [4,16,17]. Cadmium could also cause distortion to cellular membranes structure and function [3,18,19]. An increase in MDA is often used as an oxidative stress marker in physiological assays [20]. Cd stress increased lipid peroxidation in the form of MDA, and it enhanced the hydrogen peroxide content, electrolyte leakage, and proline contents in the leaves [21]. Thus, the present study measured the root relative elongation and malondialdehyde content as the indicator of Cd toxicity to the root. Our results demonstrated that the influence of Cd on the root elongation and membrane lipid peroxidation in the species with the closed-type RAM was significantly greater than in those with an open-type RAM, hence determining their difference in tolerance to cadmium stress. It also indicates that both relative root elongation and relative increase rate MDA could be used as physiological markers for resistance to Cd. Of these, the relative root elongation is more sensitive to Cd treatment. However, the relative elongation of roots couldn’t reflect plant shoot resistance to Cd since the absorption and transport mechanism of Cd is different between roots and shoots.

RBCs are considered to be the front of plant response and defense to stresses [21]. The number of RBCs and their mucilage could form a protective sheath at the root tip, which prevented Al from diffusing into the root tip, thereby alleviating Al stress in the plant root tip [22,23]. The number of RBCs upon Cd exposure decreased significantly only in peas but not in other species tested. However, the survival rate of RBCs was significantly decreased in all species upon Cd treatment. These results were similar to the reports about the effects of aluminum on the RBCs of pea and barley [13,24].

Under stress conditions, such as diseases, aluminum toxicity, or high temperature, RBCs could change gene expression and release anthocyanin, antibiotics, specific enzymes, sugars, and other chemicals into the extracellular space. Depending on the type of stress, such secretion could inhibit or promote the growth of soil-dwelling microorganisms as well as modulate the environmental conditions in the rhizosphere [11,25,26]. One of the examples of such modulation is RBCs protection of root tips from aluminum toxicity [22].

In the present study, we found that the species with an open-type RAM were more tolerant to Cd stress than the ones with closed-type RAM (as indicated by their survival rates). It is plausible to suggest that species with an open-type RAM generated more RBCs that could play important biological roles in protecting against Cd poisoning. The mechanistic basis of this protection should be a subject of a separate investigation.

## 5. Conclusions

This study showed that the number and viability of RBCs were significantly higher in the species possessing open RAM organization, and the plants with the open-type RAM are more tolerant to Cd than their closed-RAM counterparts. This difference could be due to the difference in the absolute number of RBCs (larger in open-RAM species) that may operate as buffers protecting sensitive cells in the root apex against Cd toxicity.

## Figures and Tables

**Table 1:** The effects of Cd treatments on root elongation of various species/genotypes.

Variety	Control	0.25 mmol/L CdCl_2_		0.5 mmol/L CdCl_2_		1 mmol/L CdCl_2_		2 mmol/L CdCl_2_	
	Root Length (cm)	Root Length (cm)	RRL (%)	Root Length (cm)	RRL (%)	Root Length (cm)	RRL (%)	Root Length (cm)	RRL (%)
ZW5	4.35 ± 0.08a	4.29 ± 0.07a	98.62	3.57 ± 0.05b	82.07	2.73 ± 0.04c	62.76	2.59 ± 0.04d	59.54
ZW6	4.26 ± 0.06a	3.94 ± 0.05b	92.49	3.54 ± 0.06c	83.10	2.57 ± 0.03d	60.33	2.40 ± 0.04e	56.34
HY1	4.35 ± 0.05a	3.88 ± 0.05b	89.20	3.00 ± 0.03c	68.97	2.71 ± 0.04d	62.30	2.62 ± 0.03e	60.23
JMT	3.94 ± 0.06a	3.66 ± 0.04b	92.89	2.87 ± 0.04c	72.84	2.46 ± 0.03d	62.44	2.32 ± 0.04e	58.88
DFH	2.22 ± 0.03a	1.85 ± 0.02b	83.33	1.45 ± 0.03c	65.32	1.28 ± 0.03d	57.66	1.18 ± 0.02e	53.15
YF	2.15 ± 0.07a	1.80 ± 0.04b	83.72	1.41 ± 0.03c	65.58	1.29 ± 0.02d	60.00	1.15 ± 0.03e	53.49
GJ1	3.03 ± 0.06a	2.62 ± 0.07b	86.47	2.25 ± 0.05c	74.26	1.81 ± 0.07d	59.74	1.64 ± 0.07e	54.13
ZJ5	1.52 ± 0.08a	1.31 ± 0.06b	86.18	1.14 ± 0.02c	75.00	0.88 ± 0.06d	57.89	0.82 ± 0.08e	53.95

Note: RRL = Relative root length. Data are mean ± SD (n = 30). Data labeled with different letters is significantly different at *p* < 0.05 level (Duncan’s test).

**Table 2:** The effects of Cd treatments on MDA contents in roots of various species/genotypes.

Variety	Control	0.25 mmol/L CdCl_2_		0.5 mmol/L CdCl_2_		1 mmol/L CdCl_2_		2 mmol/L CdCl_2_	
	MDA Content (μmol/g·FW)	MDA Content (μmol/g·FW)	Relative Increasing Rate (%)	MDA Content (μmol/g·FW)	Relative Increasing Rate (%)	MDA Content (μmol/g·FW)	Relative Increasing Rate (%)	MDA Content (μmol/g·FW)	Relative Increasing Rate (%)
ZW5	7.81 ± 0.94e	9.68 ± 0.81d	23.94	12.95 ± 0.73c	65.81	18.78 ± 1.71a	140.46	16.33 ± 1.68b	109.09
ZW6	7.63 ± 0.61e	10.38 ± 0.78d	36.04	12.96 ± 0.28c	69.86	19.01 ± 0.48a	149.15	17.52 ± 1.12b	129.62
HY1	5.89 ± 0.56e	6.75 ± 0.86d	14.60	9.86 ± 0.91c	67.40	12.52 ± 0.21b	112.56	14.32 ± 0.33a	143.12
JMT	7.30 ± 0.24e	10.54 ± 0.42d	44.38	13.85 ± 0.53c	89.73	16.69 ± 0.97b	128.63	21.58 ± 0.81a	195.62
DFH	5.69 ± 0.48e	8.45 ± 0.17d	48.51	10.47 ± 0.86c	84.01	12.53 ± 0.75b	120.21	15.56 ± 0.91a	173.46
YF	4.95 ± 0.74e	6.52 ± 0.15d	31.72	10.55 ± 0.64c	113.13	15.59 ± 0.66a	214.95	14.19 ± 0.74b	186.67
GJ1	5.56 ± 0.45d	7.56 ± 0.14c	35.97	9.62 ± 0.81bc	73.02	15.66 ± 0.87a	181.65	10.64 ± 0.15b	91.37
ZJ5	3.40 ± 0.51e	4.47 ± 0.44d	31.47	6.50 ± 0.72c	91.18	7.51 ± 0.71b	120.88	8.54 ± 0.53a	151.18

Note: Data are mean ± SD (n = 30). Data labeled with different letters is significantly different at *p* < 0.05 level (Duncan’s test).

## Data Availability

Not applicable.
